# PET Tumour Segmentation in Head and Neck Cancer: Reproducibility, Reader Dependence and Concordance with CT-Defined Tumour Volume in a 154-Patient Cohort

**DOI:** 10.3390/cancers18193214

**Published:** 2026-10-05

**Authors:** Anne-Sophie Pirson, Gilles Delahaut, Raphaël Georis, Stéphanie Deheneffe, Sébastien Van der Vorst, Ken Kudura

**Affiliations:** 1 Department of Nuclear Medicine, CHU UCL Namur, 5530 Yvoir, Belgium; anne-sophie.pirson@chuuclnamur.uclouvain.be; 2 Department of Otolaryngology—Head and Neck Surgery, CHU UCL Namur, 5530 Yvoir, Belgium; 3 Department of Biostatistics, CHU UCL Namur, 5530 Yvoir, Belgium; raphael.georis@chuuclnamur.uclouvain.be; 4 Department of Radiation Oncology, CHU UCL Namur, 5000 Namur, Belgium

**Keywords:** head and neck cancer, metabolic tumour volume, PET/CT segmentation, MIM Maestro, reproducibility, gross tumour volume

## Abstract

**Purpose:** Volumetric PET biomarkers carry substantial prognostic value in head and neck squamous cell carcinoma (HNSCC), yet their clinical implementation is hampered by segmentation heterogeneity. A fundamental and unresolved question is which segmentation method is most suitable for routine clinical use, both for a reader experienced in PET segmentation and for a reader without PET segmentation experience, such as an ENT surgeon or a radiation oncologist. This study addressed this question by assessing the intra- and interobserver reproducibility of four PET segmentation methods in MIM Maestro Software Version 7.4.2, identifying tumour-related determinants of variability, and examining concordance between PET-derived metabolic tumour volume (MTV) and CT-based gross tumour volume (GTV). **Methods:** Two readers with contrasting PET segmentation experience, a nuclear medicine physician experienced in PET segmentation (R1) and an ENT surgeon without prior PET segmentation training (R2), applied four methods to the primary tumours of 154 patients with HNSCC (oral cavity, oropharynx, hypopharynx or larynx) treated with definitive (chemo)radiotherapy between 2010 and 2021: fixed absolute thresholds at SUV 3.0 and 4.0, a relative threshold at 41% of the maximum standardised uptake value (SUVmax), and a gradient edge-detection algorithm (PET Edge+). R1 segmented twice with a washout of at least 4 weeks; R2 performed one session and delineated CT-GTV in the 85 patients whose PET/CT-derived CT served directly as the radiotherapy planning scan. The second session of R1 served as the reference for all interobserver and MTV–GTV analyses. Agreement was assessed by ICC (two-way mixed model), coefficient of variation, repeatability coefficient and Bland–Altman analysis; determinants of variability by a multivariable linear mixed-effects model with a random intercept per patient. **Results:** All four methods demonstrated excellent intraobserver (ICC 0.993 to >0.999) and interobserver (ICC 0.989–0.996) reproducibility. SUV3.0 generated significantly more relative error than SUV41% (β = +3.1%; 95% CI 0.89, 5.3). PET Edge+ was the only method for which a significant method × reader interaction was observed in this reader pair: the reader without PET segmentation experience generated more error than the reader experienced in PET segmentation (β = +7.1%; 95% CI 3.9, 10), an effect concentrated in smaller tumours (below approximately 15 mL); in the 85-patient GTV subset, where tumours were larger on average, the interobserver ICC for PET Edge+ was 0.997. GTV concordance was good for three of four methods (ICC 0.808–0.895); SUV41% showed only moderate concordance (ICC 0.572). **Conclusions:** All four methods provided excellent volumetric reproducibility within a single clinical software platform, but they differed in reader dependence and in agreement with CT-defined GTV. Rather than supporting a universal segmentation standard, these findings suggest that the method should be selected according to its intended application, pending multicentre confirmation.

## Simple Summary

The volume of metabolically active tumour measured on PET/CT scans helps to predict outcome in head and neck cancer, but different ways of outlining the tumour on the images can give different results. We compared four outlining methods available in a widely used clinical software in 154 patients with head and neck squamous cell carcinoma. Each method was applied by a nuclear medicine physician experienced in PET segmentation and by an ENT surgeon without such experience. All four methods produced highly reproducible tumour volumes. However, only the semi-automatic gradient-based method was sensitive to the reader’s experience, and only in small tumours, whereas agreement with the tumour volume outlined on CT was best for the fixed threshold at SUV 4.0. No single method is best for every purpose; the choice should depend on how the measurement will be used.

## 1. Introduction

Head and neck cancers (HNC) represent a heterogeneous group of malignancies arising from the mucosal surfaces of the oral cavity, oropharynx, hypopharynx, and larynx, characterised by complex anatomy and highly variable biological behaviour [1,2]. Squamous cell carcinoma accounts for the large majority of these tumours (head and neck squamous cell carcinoma, HNSCC) and is the only histological type addressed in the present study. 18F-FDG PET/CT plays a critical role in initial staging and therapeutic management of these tumours, providing complementary functional and morphological information that influences clinical decisions in a meaningful proportion of patients [3,4].

Beyond visual interpretation, quantitative PET-derived biomarkers, particularly metabolic tumour volume (MTV) and total lesion glycolysis (TLG), have demonstrated significant prognostic value, with meta-analyses supporting their association with overall and disease-free survival in HNC patients [5,6].

However, the clinical implementation of volumetric PET biomarkers in head and neck oncology remains limited by a critical unresolved issue: the lack of standardisation of tumour segmentation methods [7,8]. This limitation is particularly relevant in HNC, where primary lesions are often small, heterogeneous, and located in anatomical regions with high physiological FDG uptake, including salivary glands, muscles, and lymphoid tissue (Waldeyer’s ring), making tumour delineation technically challenging and subject to substantial intra- and interobserver variability [7].

Previous studies have demonstrated that MTV and TLG are highly dependent on the segmentation approach used, with variations that may directly influence quantitative results and patient risk stratification [8,9]. Nevertheless, most available data compare segmentation methods across different software platforms or rely on experimental algorithms, which hampers their direct translation into routine clinical practice. No study has jointly evaluated reproducibility, its tumour-related determinants, and agreement with CT-based gross tumour volume (GTV) within the same large cohort and a single widely deployed clinical software environment. Moreover, the practical question of whether reliable PET segmentation can be performed by readers without dedicated PET segmentation experience (of direct relevance to multidisciplinary head and neck oncology teams) has not been formally addressed.

This study therefore pursues three progressive objectives. The **primary objective** was to assess and compare the intra- and interobserver reproducibility of four PET-based segmentation methods in MIM Software Version 7.4.2 in 154 patients with primary HNSCC. As a **secondary objective**, we investigated whether differences between segmentation methods were associated with tumour-related characteristics, including volume, hypermetabolism, and primary site. As a **tertiary objective**, we examined the quantitative relationship between PET-derived MTV and the CT-defined GTV. Together, these three objectives serve a single general aim: to determine which PET segmentation method available in a widely used clinical software platform is best suited to routine clinical use in HNSCC, according to its reproducibility, its dependence on PET segmentation experience and its agreement with CT-defined tumour volume.

## 2. Materials and Methods

### 2.1. Study Design and Patients

This retrospective single-centre study was conducted at CHU UCL Namur (Belgium). All patients treated with definitive radiotherapy or chemoradiotherapy at the Radiation Oncology Department of CHU UCL Namur between 1 January 2010 and 31 December 2021 were screened (n = 614). Inclusion criteria were: biopsy-confirmed infiltrative HNSCC of the oral cavity, oropharynx, hypopharynx or larynx; a multidisciplinary tumour board recommendation for radiotherapy or chemoradiotherapy, according to local guidelines; and the availability of diagnostic and planning CT scans at CHU UCL Namur. Exclusion criteria were: a previous malignancy within 5 years (except basal cell carcinoma, in situ cervical carcinoma, or T1 glottic or lip tumours completely resected with R0 margins); prior tumour resection or neck dissection; primary tumours not measurable on diagnostic CT (e.g., cT1 glottic tumours or lesions obscured by metal artefacts); primary tumours of the nose, paranasal sinuses, oesophagus, salivary glands or nasopharynx; synchronous second cancers; distant metastases; and the absence of a pre-treatment 18F-FDG PET/CT. Only squamous cell carcinomas were eligible; no other histological type was included, and poorly distinguishable primary lesions that could not be measured on CT were therefore not included. A total of 154 patients met all inclusion criteria and no exclusion criterion and formed the final study cohort (Figure 1). Human papillomavirus (p16) status was available only inconsistently for oropharyngeal tumours, because p16 immunohistochemistry was not systematically performed during the early part of the screening period; it was therefore not used as an analysis variable. Of the 154 patients included, 85 (55%) had their PET/CT-derived CT used directly as the radiotherapy planning scan without a separate dedicated planning acquisition; only this subgroup was eligible for the MTV–GTV concordance analysis (Objective 3). In the remaining 69 patients, the planning CT was acquired at a variable interval after the PET/CT, introducing an uncontrolled temporal confound that would render volumetric comparison between the two modalities uninterpretable. A formal comparison of the demographic and tumoral characteristics of the 85 included patients and the 69 excluded patients is provided in Table 1.

Ethics approval was obtained (reference: CE Mont-Godinne: 69/2023; date of approval: 27 April 2023). This study was conducted and reported in accordance with the STROBE guidelines.

### 2.2. PET/CT Acquisition

All 18F-FDG PET/CT examinations were performed on a single PET/CT system (Gemini TF 16, Philips Healthcare, Best, The Netherlands) throughout the study period. Patients fasted for at least 6 h, and blood glucose had to be below 180 mg/dL before injection. An activity of 3.3–3.5 MBq/kg of 18F-FDG was administered intravenously, and images were acquired 60 min after injection with the arms positioned alongside the body. The head and neck region was covered by 2 or 3 bed positions of 150 s each. PET data were reconstructed with the vendor BLOB-OS-TF algorithm (3 iterations, 33 subsets), including CT-based attenuation correction, scatter correction and time-of-flight information, with a 288 × 288 matrix, a slice thickness of 2 mm and a voxel size of 2.0 mm. The CT component consisted of a topogram followed by a helical acquisition (120 kV; 50–250 mA per slice; slice thickness 3–5 mm). This CT was therefore a diagnostic-quality helical acquisition rather than a low-dose attenuation-correction CT only. In the 85 patients of the MTV–GTV concordance cohort, the CT was acquired with iodinated contrast enhancement and was used directly as the radiotherapy planning CT and for CT-GTV delineation, whereas in the remaining 69 patients a separate dedicated radiotherapy planning CT was acquired.

### 2.3. Readers and Segmentation

**Reader 1 (R1):** Nuclear medicine physician (20+ years of clinical experience) with dedicated PET segmentation experience. R1 performed two independent segmentation sessions (minimum 4-week washout; no access to prior contours) for intraobserver analysis. The second session of R1 (R1 Session 2) was defined, before the analyses were performed, as the reference reading for all interobserver comparisons with R2 and for all MTV–GTV concordance analyses.

**Reader 2 (R2):** Board-certified ENT surgeon with recognised HNC anatomical expertise but without prior formal PET segmentation training. R2 performed one independent session for interobserver analysis, and separately delineated CT-GTV on co-registered CT images (standard soft-tissue windowing) in the 85 eligible patients under the supervision of an experienced radiation oncologist. Both readers were fully blinded to each other’s contours and to clinical data and outcome throughout.

Several measures were taken to limit selection and observer-related bias. Selection bias was limited by screening all patients treated with definitive (chemo)radiotherapy over a 12-year period against predefined eligibility criteria (Figure 1), and by formally comparing the patients included in and excluded from the GTV concordance analysis (Table 1). Observer-related bias was limited by blinding both readers to each other’s contours and to clinical data and outcome, by separating the two R1 sessions by a washout period of at least 4 weeks without access to previous contours, by applying the three threshold methods in a fully automated manner without manual editing, by documenting every manual correction of PET Edge+ contours, and by entrusting all statistical analyses to an independent biostatistician who was not involved in segmentation.

The four methods evaluated in this study represent the PET segmentation approaches routinely used at our institution within MIM Maestro^®^ Version 7.4.2 (MIM Software Inc., Cleveland, OH, USA), our clinical software platform, and are broadly representative of the major methodological categories described in the literature: fixed relative thresholds, fixed absolute thresholds, and gradient-based algorithms [10]. The restriction to a single software platform was deliberate: it removes inter-platform variability as a confound and ensures that the findings are directly applicable to the workflow in which these methods will actually be used.

Four segmentation methods were evaluated (Figure 2).

**SUV3.0:** Automated absolute threshold at 3.0 for SUVmax.

**SUV4.0:** Automated absolute threshold at 4.0 for SUVmax.

**SUV41%:** Automated relative threshold at 41% for SUVmax.

**PET Edge+:** Semi-automated gradient edge-detection algorithm, initiated by operator seed placement and converging on the steepest SUV gradient at the tumour boundary [10]. Unlike the threshold-based methods, which were applied without manual correction to assess their fully automated use, PET Edge+ was used with its full native functionality, including the manual grow/shrink correction tools, which the reader could apply when the automated contour required adjustment. The use of these corrections was documented for each patient in the study database (REDCap) and reviewed retrospectively.

**Analytical reference categories.** SUV41% was used as the reference category for all regression analyses across the three study objectives to ensure methodological consistency throughout the manuscript. This method was selected because it demonstrated the highest interobserver reproducibility and produced the smallest MTV estimates in the present cohort, providing a stable baseline against which differences between segmentation methods could be expressed. Although fixed absolute thresholds such as SUV3.0 are widely reported in the HNC literature [7], and although volumetric MTV has been evaluated prospectively as a prognostic biomarker in the RTOG 0522 trial [11], the reference category for the regression analyses was chosen to ensure internal consistency across all study objectives rather than to reflect historical convention. The choice of reference category was therefore made solely for model parameterisation and does not imply clinical superiority of SUV41%. Importantly, the principal study conclusions are based on absolute agreement measures (ICC and Bland–Altman analyses) together with the relative ranking of the regression coefficients, both of which are independent of the selected regression reference.

**Figure 2:**
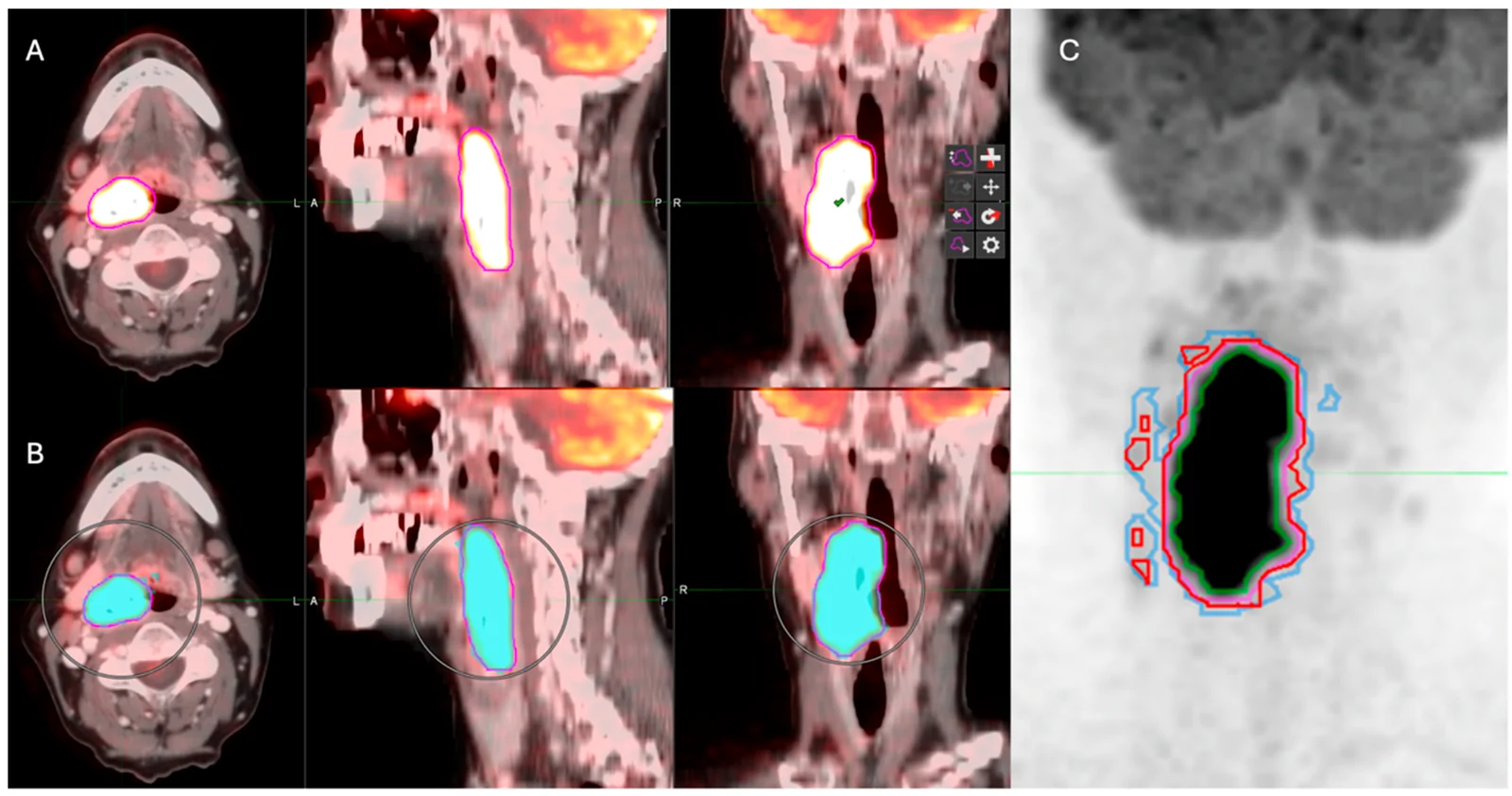
Representative example of the four PET segmentation methods implemented in MIM software Version 7.4.2 in a patient with stage III p16-positive oropharyngeal squamous cell carcinoma according to the 8th edition of the AJCC staging system. (**A**) PET Edge+ gradient-based segmentation. (**B**) Regions of interest used for threshold determination. (**C**) Maximum intensity projection displaying segmentation contours: PET Edge+ (pink), SUV 4 (red), SUV 3 (blue), and 41% SUVmax threshold (green).

### 2.4. Statistical Analysis

Statistical analyses were performed in R (v4.5; June 2026) by an independent biostatistician. Agreement was quantified by: (a) the intraclass correlation coefficient (ICC; two-way mixed model, absolute agreement) with 95% CI derived from the Fisher z-transformation; (b) Bland–Altman analysis in both absolute (mL) and relative (%) units, with 95% limits of agreement; (c) the within-subject coefficient of variation (CV, %); and (d) the repeatability coefficient (RC, mL), i.e., the value below which the absolute difference between two measurements of the same tumour is expected to lie in 95% of cases (1.96 × the standard deviation of the paired differences). ICC grading: ≥0.90 = excellent; 0.75–0.89 = good; 0.50–0.74 = moderate; <0.50 = poor (Objective 1) [12]. Because the ICC increases with between-subject variance, which is large in a cohort whose tumour volumes span approximately two orders of magnitude, ICC values were interpreted together with the CV, the RC and the Bland–Altman limits of agreement, and are reported to three decimal places. Intraobserver agreement compared R1 Session 1 with R1 Session 2; interobserver agreement compared R1 Session 2 with R2. For Objective 2, the absolute relative MTV error with respect to R1 Session 2 was modelled with a multivariable linear mixed-effects model (R package lme4) including segmentation method, reader (R1 Session 1 vs. R2), the method × reader interaction, tumour volume, SUVmax and primary site as fixed effects, and a random intercept per patient to account for the non-independence of the 1232 observations (154 patients × 4 methods × 2 readings). For Objective 3, concordance between MTV (R1 Session 2) and CT-GTV was assessed using ICC, Bland–Altman plots, and a multivariable regression model of the same structure on the absolute relative MTV–GTV discordance. All *p*-values are two-sided (significance threshold: 0.05).

## 3. Results

### 3.1. Patient Characteristics

Of the 614 patients screened, 154 patients with primary HNSCC were included (Figure 1). The cohort was predominantly male (79%), with a median age of 61 years (IQR 56–66). Tobacco exposure was documented in 85%. The oropharynx was the most frequent primary site (54%), followed by hypopharynx (25%), larynx (16%), and oral cavity (5%). Most patients received concurrent chemoradiotherapy (86%). Median SUVmax was 16 (IQR 12–20). MTV varied substantially by method: SUV41% had the smallest volumes (median 7 mL, IQR 3–13), PET Edge+ intermediate values (9 mL, 5–18), SUV4.0 moderate values (12 mL, 5–23), and SUV3.0 the largest (17 mL, 9–32). CT-GTV was available in 85 patients (55%), with a median of 15 mL (IQR 7–25) (Table 2).

**Table 1:** Comparison of baseline characteristics between the GTV-eligible subset (n = 85, whose PET/CT-derived CT was used directly as the radiotherapy planning scan) and excluded patients (n = 69). *p*-values from Fisher’s exact test (categorical) or Mann–Whitney U (continuous). No other baseline characteristic differed significantly between groups. † Global *p*-value for the distribution of primary sites. ‡ Difference expected by design (definition of the two subgroups).

Characteristic	GTV Subset n = 85	Excl. from GTV n = 69	*p*-Value
***Demographics***			
Age—median (IQR)	62 (56, 66)	60 (55, 64)	0.27
Sex—male, n (%)	69 (81%)	52 (75%)	0.44
Active/former smoker, n (%)	74 (87%)	58 (84%)	0.62
Alcohol ≥ 1, n (%)	41 (49%)	39 (57%)	0.38
***Primary tumour site***			
Oropharynx, n (%)	45 (53%)	38 (55%)	0.72 †
Hypopharynx, n (%)	20 (24%)	18 (26%)	
Larynx, n (%)	14 (16%)	11 (16%)	
Oral cavity, n (%)	6 (7.1%)	2 (2.9%)	
***Oncological staging***			
Stage IVa–IVb, n (%)	66 (78%)	50 (72%)	0.48
cT3–T4, n (%)	60 (71%)	44 (64%)	0.41
cN2–N3, n (%)	54 (64%)	50 (72%)	0.31
***Treatment***			
Chemoradiotherapy, n (%)	74 (87%)	58 (84%)	0.62
***PET/CT parameters***			
SUVmax—median (IQR)	15 (12, 21)	16 (13, 19)	0.58
PET–planning CT interval (days)	0 (0, 0)	15 (7, 28)	<0.001 ‡

**Table 2:** Demographic, clinical and PET/CT characteristics of the full study cohort (N = 154). IQR = interquartile range. Alcohol score: 0 = none; 1 = occasional; 2 = regular; 3 = daily/heavy. Percentages are calculated among patients with available data for each variable.

Characteristic	N = 154
***Demographics***	
Age—median (IQR), years	61 (56–66)
Sex—male, n (%)	121 (79%)
Active smoker, n (%)	93 (60%)
Former smoker, n (%)	39 (25%)
Non-smoker, n (%)	12 (7.8%)
Unknown smoking status, n	10
Alcohol use (score ≥ 1), n (%)	80 (52%)
Unknown alcohol status, n	1
***Primary tumour site***	
Oropharynx, n (%)	83 (54%)
Hypopharynx, n (%)	38 (25%)
Larynx, n (%)	25 (16%)
Oral cavity, n (%)	8 (5.2%)
***Oncological staging (AJCC 8th ed.)***	
Stage I, n (%)	3 (2.0%)
Stage II, n (%)	13 (8.6%)
Stage III, n (%)	19 (13%)
Stage IVa, n (%)	78 (52%)
Stage IVb, n (%)	38 (25%)
Unknown stage, n	3
***Clinical TNM***	
cT1, n (%)	9 (5.8%)
cT2, n (%)	41 (27%)
cT3, n (%)	44 (29%)
cT4, n (%)	60 (39%)
cN0, n (%)	39 (25%)
cN1, n (%)	23 (15%)
cN2 (a + b +c ), n (%)	59 (38%)
cN3, n (%)	33 (21%)
cM0, n (%)	152 (100%)
Unknown cM, n	2
***Treatment***	
Concurrent chemoradiotherapy, n (%)	132 (86%)
Radiotherapy alone, n (%)	22 (14%)
***PET/CT quantitative parameters (R1, Session 2)***	
SUVmax—median (IQR)	16 (12–20)
SUVmean—median (IQR)	8.2 (6.0–10.2)
SUV41% MTV—median (IQR), mL	7 (3–13)
PET Edge+ MTV—median (IQR), mL	9 (5–18)
SUV4.0 MTV—median (IQR), mL	12 (5–23)
SUV3.0 MTV—median (IQR), mL	17 (9–32)
***Morphological reference (CT-GTV, R2 delineation)***	
CT-GTV available, n (%)	85 (55%)
CT-GTV—median (IQR), mL	15 (7–25)

### 3.2. Intraobserver Reproducibility

Intraobserver ICC values (R1 Session 1 vs. Session 2) were excellent across all four methods on the full cohort: 0.993 for SUV3.0 (95% CI 0.990, 0.995), 0.994 for SUV4.0 (0.992, 0.996), 0.994 for SUV41% (0.992, 0.996), and >0.999 for PET Edge+ (>0.999, >0.999). Within-subject CVs were 8.1%, 7.9%, 7.4% and 1.7%, and RCs 5.23, 3.71, 1.97 and 0.68 mL, respectively (Table 3). Bland–Altman analysis confirmed negligible bias; 95% limits of agreement in absolute units were widest for SUV3.0 (+5.15 to −5.34 mL) and narrowest for PET Edge+ (+0.72 to −0.63 mL) (Figure 3).

### 3.3. Interobserver Reproducibility

Interobserver ICC values (R1 Session 2 vs. R2) were similarly high for all methods: 0.994 for SUV3.0 (95% CI 0.991, 0.995), 0.994 for SUV4.0 (0.992, 0.996), 0.996 for SUV41% (0.994, 0.997), and 0.989 for PET Edge+ (0.984, 0.992). Within-subject CVs were 7.3%, 7.5%, 6.2% and 11.3%, and RCs 4.78, 3.56, 1.64 and 4.46 mL, respectively. The largest intraobserver-to-interobserver attenuation was observed for PET Edge+ (ICC > 0.999 to 0.989; CV 1.7% to 11.3%; RC 0.68 to 4.46 mL), whereas for the three threshold methods the interobserver CVs and RCs were similar to or lower than the intraobserver values (Figure 4 and Figure 5; Table 3). Manual grow/shrink corrections of PET Edge+ contours were applied in approximately 30 of the 154 patients (≈19%), and their use was discordant between the two readers in 9 of these patients.

### 3.4. Determinants of Segmentation Variability

The variability regression (linear mixed-effects model with a random intercept per patient; absolute relative MTV error with respect to R1 Session 2; reference: SUV41%; N = 1232 observations from 154 patients) identified three independent predictors. First, SUV3.0 generated significantly more absolute relative error than SUV41% (β = +3.1%; 95% CI 0.89, 5.3), while SUV4.0 (+0.77%) and PET Edge+ (−0.05%) did not differ significantly. Because this estimate was obtained from a model that accounts for within-patient clustering, it is not an artefact of treating repeated observations as independent. Second, higher SUVmax (β = −0.32/unit) and larger tumour volume (β = −0.09/mL) independently reduced error. Third, oropharyngeal primaries showed the highest error rates; hypopharyngeal (β = −3.8%) and laryngeal (β = −3.2%) tumours were significantly more reproducible. The main effect of the reader without PET segmentation experience (R2) was non-significant (β = −0.15%). The only significant interaction was PET Edge+ × reader without PET segmentation experience (β = +7.1%; 95% CI 3.9, 10). No analogous interaction was detected for SUV4.0 (+0.70%) or SUV3.0 (+0.75%) (Figure 6 and Figure 7; Table 4).

#### The ~15 mL Threshold and Its Relevance to Cohort Composition

Visual inspection of the regression-predicted error curves suggested that, above a volume of approximately 15 mL, the predicted errors of PET Edge+ and SUV4.0 converge regardless of PET segmentation experience; this is a graphical observation, and 15 mL was not formally tested as a cut-off. The interaction effect that distinguishes the two methods appears to be largely confined to tumours below this volume. The clinical relevance of this observation depends on what proportion of HNSCC tumours fall below 15 mL in a typical staging cohort. In the present cohort, the median PET Edge+ volume was 9 mL (IQR 5–18), and approximately 60% of tumours had a PET Edge+ volume below 15 mL in this cohort (Figure 7).

### 3.5. Exploratory Analysis: Reproducibility in Tumours > 5 mL

To further characterise the volume-dependence of reproducibility, an exploratory analysis was conducted on the subset of tumours with PET Edge+ > 5 mL. Intraobserver ICC values in this subset remained excellent and were largely unchanged: SUV3.0 0.992, SUV4.0 0.992, SUV41% 0.995, PET Edge+ > 0.999. Interobserver ICC values (R1 Session 2 vs. R2) were: SUV41% 0.997, SUV4.0 0.993, SUV3.0 0.992, and PET Edge+ 0.986. On the 85-patient GTV subset specifically (where median PET Edge+ was 11 mL), the interobserver ICC for PET Edge+ was 0.997 (CV 5.4%; RC 2.09 mL, compared with 11.3% and 4.46 mL in the full cohort), the second highest in that subgroup after SUV41% (0.999), suggesting that the penalty associated with the absence of PET segmentation experience is attenuated in the volume range typical of GTV-eligible tumours. These findings are consistent with the interpretation that the interaction effect identified in the primary regression is driven predominantly by small-volume tumours.

### 3.6. MTV Versus CT-GTV

ICC between R1 MTV (Session 2) and CT-GTV was good for three methods: SUV4.0 (0.895; 95% CI 0.858, 0.922), PET Edge+ (0.846; 0.734, 0.904), and SUV3.0 (0.808; 0.658, 0.883). SUV41% showed only moderate concordance (0.572; 0.050, 0.791). In the GTV discordance regression (reference: SUV41%), SUV4.0 significantly reduced absolute percentage error (β = −16%; 95% CI −25, −8.0) as did PET Edge+ (β = −10%; −18, −1.7); SUV3.0 did not differ significantly from SUV41% (+2.2%). Bland–Altman analysis showed: SUV3.0 positive bias (+7.2 mL; LoA −17.41 to +30.58 mL); SUV4.0 near-zero bias (−1.2 mL; LoA −17.72 to +15.57 mL); SUV41% negative bias (−10.2 mL; LoA −28.18 to +7.18 mL); PET Edge+ negative bias (−5.2 mL; LoA −21.16 to +12.1 mL) (Figure 8 and Figure 9; Table 5).

## 4. Discussion

### 4.1. A Shared Foundation: Excellent Reproducibility Across All Methods

The primary finding of this study is that all four segmentation methods yielded excellent intra- and interobserver reproducibility for MTV across 154 patients with primary HNSCC, with intraobserver ICCs ≥ 0.993 and interobserver ICCs ≥ 0.989. Because the ICC is influenced by between-subject variance, the very high values observed in this heterogeneous volume distribution (approximately 1 to 100 mL) should not be interpreted in isolation, and small differences between ICCs in this range should not be over-interpreted. The CV, the RC and the Bland–Altman limits of agreement therefore provide complementary information on measurement error: within-subject CVs ranged from 1.7% to 8.1% (intraobserver) and from 6.2% to 11.3% (interobserver), and RCs from 0.68 to 5.23 mL. These estimates compare favourably with published data, although direct comparison is limited by differences in volume distribution, software and study design. Schinagl et al., in one of the most frequently cited comparative studies in HNC, showed that the choice of segmentation tool substantially affects PET-based target volumes [7]. Pfaehler et al. reported variable repeatability of conventional and AI-based semi-automatic segmentation approaches [13], and good intra- and inter-operator agreement of MTV has also been reported in other tumour types, including soft tissue tumours [14]. The use of a single software platform, which removes inter-platform variability, may have contributed to the high agreement observed here; this remains a plausible hypothesis, however, as it could not be directly tested in the absence of a multi-platform comparison. In contrast, inter-observer variability for PET-derived texture features in HNC substantially exceeds these values [15], further supporting the robustness of volumetric over textural PET biomarkers. The practical implication is that, within MIM Maestro Software, method choice did not materially compromise MTV reproducibility in this cohort. The clinical questions therefore shift to which method introduces the least measurement noise, which is most independent of PET segmentation experience, and which shows the closest agreement with CT-defined GTV, questions that the remaining objectives of this study address.

### 4.2. Distinct Performance Profiles: What Each Method Optimises

Three findings differentiate the methods beyond their shared reproducibility.

First, the variability regression identified a slightly higher relative error for SUV3.0 compared with SUV41% (β = +3.1%; 95% CI 0.89–5.3), whereas SUV4.0 and PET Edge+ showed no significant difference. This estimate was derived from a mixed-effects model accounting for within-patient clustering and remained statistically significant, although its magnitude is small. This finding may appear unexpected, as fixed absolute thresholds are widely used in HNC studies [7]. However, SUV3.0 systematically generated larger MTVs than SUV41% (median 17 mL vs. 7 mL), reflecting the inclusion of lower-intensity peripheral tumour uptake. Consequently, small absolute contour differences may have different relative consequences depending on the underlying tumour volume. Importantly, this result should not be interpreted as indicating poor reliability of SUV3.0: Bland–Altman analysis showed only modest absolute differences (limits of agreement: +5.15 to −5.34 mL). Rather, it highlights that relative error metrics should be interpreted in the context of the volume distribution generated by each segmentation method.

Second, PET Edge+ achieved the highest intraobserver ICC of all four methods (>0.999), the lowest intraobserver CV (1.7%) and RC (0.68 mL), and the narrowest absolute limits of agreement (+0.72 to −0.63 mL), a reflection of the deterministic nature of the gradient algorithm when seed placement by a reader experienced in PET segmentation initiates the convergence process [10]. The regression-predicted error curves for PET Edge+ and SUV4.0 appear to converge at approximately 15 mL; above this volume, the predicted performance of PET Edge+ is similar to that of SUV4.0 regardless of PET segmentation experience. Below it, however, the performance of the gradient method is sensitive to seed placement, and the reader without PET segmentation experience generated more error, as reflected by the rise in CV from 1.7% (intraobserver) to 11.3% (interobserver). In the present series, the median PET Edge+ volume across 154 patients was 9 mL (IQR 5–18), meaning that approximately 60% of tumours measured by this method fell below the 15 mL threshold. Conversely, it should be noted that the approximately 40% of tumours above 15 mL, which include the largest and most metabolically active lesions, can apparently be segmented with PET Edge+ by either reader with a performance similar to that of SUV4.0. The confirmatory analysis on the 85-patient GTV subset (where median PET Edge+ was 11 mL and interobserver ICC was 0.997) further supports this interpretation: in a subgroup with somewhat larger tumours, the performance penalty diminishes substantially. Several mechanisms may account for the volume-dependent reader sensitivity of the gradient method. In small lesions, seed placement is more consequential because the algorithm has less metabolic gradient to exploit before encountering noise or adjacent physiological uptake; in large lesions with well-defined metabolic margins, the gradient signal is strong and directionally unambiguous, making convergence largely independent of seed position. These explanations remain hypothetical and require confirmation in a multi-reader study. Differential use of the manual grow/shrink correction tools may also have contributed to inter-reader variability for PET Edge+: corrections were applied in approximately 30 patients, and their use was discordant between readers in 9. The need for manual adjustment of semi-automatic gradient-based measurements has also been examined in other tumour types [16]. A pre-specified protocol governing their use is a prerequisite for multi-reader deployment.

Third, SUV4.0 achieves the highest ICC with CT-GTV (0.895) and the smallest mean absolute bias (−1.2 mL), making it the method with the closest agreement to CT-defined GTV in this cohort. PET Edge+ and SUV3.0 show good but lower concordance (ICC 0.846 and 0.808, respectively). Differences in MTV–GTV concordance should not be interpreted as reflecting the intrinsic superiority of one segmentation method over another, as they are partly determined by the methodological characteristics of each approach and by the study protocol. SUV3.0 showed good concordance (ICC 0.808) but systematically overestimated GTV (+7.2 mL). Two factors must be distinguished here. The first is intrinsic to the threshold level: a fixed threshold of SUV 3.0 lies close to the uptake of inflamed peritumoral tissue and of adjacent physiological FDG activity, particularly in the oropharynx where uptake in Waldeyer’s ring often merges with the primary tumour, a finding consistent with the known discordance between PET-derived MTV and pathological tumour volume in squamous cell carcinoma of the oral cavity [17]. The second is methodological: the three threshold methods were deliberately applied without manual editing to evaluate their fully automated performance, whereas PET Edge+ contours could be corrected with the grow/shrink tools. This asymmetry cannot, by itself, explain the overestimation by SUV3.0, since SUV4.0 and SUV41% were applied under exactly the same fully automated protocol and showed near-zero (−1.2 mL) and negative (−10.2 mL) biases, respectively; the positive bias of SUV3.0 is therefore attributable mainly to its threshold level rather than to the absence of correction. Conversely, the asymmetry may have favoured PET Edge+ in the comparison between methods, since manual correction was applied in approximately one fifth of patients (about 30 of 154); the concordance of PET Edge+ with GTV should therefore be understood as that of a semi-automated, operator-corrected method. In routine clinical practice, manual editing would likely exclude part of this non-tumour uptake, resulting in smaller MTVs and potentially a different level of agreement with CT-GTV. PET Edge+ also demonstrated good concordance (ICC 0.846) but with a systematic negative bias (−5.2 mL), which may reflect the tendency of gradient-based segmentation to converge towards the inner metabolic boundary of smaller lesions. SUV41% showed only moderate concordance with CT-GTV (ICC 0.572) and the largest negative bias (−10.2 mL), consistent with the known tendency of relative thresholding to underestimate tumour extent in the metabolically complex head and neck region [7,17]. Adaptive thresholding approaches have been proposed to address this limitation [18], though their impact on inter-reader reproducibility and mid-treatment response prediction remains variable [19].

Taken together, these findings indicate that the choice of segmentation method should not be driven solely by reproducibility, as all approaches performed well in this regard, but also by reader dependence and by the agreement required with CT-defined tumour volume. Because MTV values varied substantially by method (median 7–17 mL in this cohort), the same tumour can be assigned markedly different volumes depending on the method, and MTV cut-offs derived with one method cannot be transferred to another. This is relevant for prognostic applications: pre-therapeutic MTV has demonstrated independent prognostic value in pharyngeal and head and neck cancers treated with definitive (chemo)radiotherapy [9,20], and as a predictor of radiotherapy response [21], and methodological consistency is a prerequisite for meaningful cross-study comparison of MTV-based prognostic data [22]. Cross-study comparability was not directly evaluated here, and the present work was deliberately confined to reproducibility, reader dependence and agreement with CT-defined tumour volume; the prognostic performance of the four segmentation methods in this cohort is the subject of a separate study, currently under review. The distinct performance profiles of the four methods are summarised in Table 6.

Operator dependence should not, however, be regarded as an unavoidable trade-off. In the present study, the only method showing a significant reader × method interaction was the one requiring manual seed placement, whereas fully automated deep-learning segmentation can substantially reduce direct reader input. As recently demonstrated in other PET settings, such as U-Net-based segmentation with transfer learning for [18F]F-DOPA PET in paediatric gliomas, deep-learning approaches can delineate small lesions surrounded by physiological uptake, conditions comparable to those identified here as problematic for gradient-based segmentation [23]; such approaches have not, however, been validated in FDG PET of HNSCC. They could, in principle, combine the reproducibility of automated thresholds with a closer agreement with CT-defined tumour volume. Their use in HNSCC will nevertheless require training on large annotated datasets, external validation across scanners and centres, and demonstration of consistency with established MTV-based prognostic data before they can replace the methods evaluated here.

**Table 6:** Summary of the performance profile of each segmentation method within MIM Maestro Software across the three analytical dimensions evaluated in this retrospective single-centre study. The table reflects the performance observed in this cohort and does not imply the universal superiority of any method; fully automated deep-learning segmentation, not evaluated here, may reduce operator dependence.

Performance Dimension	Preferred Method in This Study	Key Strength	Key Limitation
Consistency with published HNC literature	SUV3.0	Fixed absolute thresholds widely reported in HNC studies	Largest volumes; GTV overestimation without manual editing; cross-study comparability not directly tested.
Inter-reader consistency	SUV41%	Highest interobserver ICC and lowest interobserver CV and RC; no significant reader × method interaction in this reader pair	Systematic GTV underestimation; moderate GTV concordance.
Agreement with CT-GTV	SUV4.0	Best agreement with CT-GTV; no significant reader × method interaction in this reader pair	Higher intraobserver CV and RC than PET Edge+.
Intra-reader precision (reader experienced in PET segmentation)	PET Edge+	Narrowest limits of agreement; lowest intraobserver CV and RC	Only method with a significant reader × method interaction (smaller tumours); protocol for grow/shrink corrections required.

This study has several limitations. First, it was a retrospective single-centre study performed on a single PET/CT system and within a single segmentation software platform. Although this design removed inter-scanner and inter-platform variability as confounders, the reproducibility estimates and method rankings reported here cannot be assumed to apply to other scanners, reconstruction settings or segmentation software, and they were not evaluated in EANM/EARL-harmonised multicentre datasets [22]. Second, despite systematic screening against predefined criteria, selection bias cannot be excluded. The GTV concordance analysis was restricted to the 85 patients whose PET/CT-derived CT served directly as the radiotherapy planning scan, a deliberate decision to avoid an uncontrolled temporal confound; the formal comparison with the 69 excluded patients showed no significant difference in age, sex, tobacco use, treatment modality, primary site, SUVmax or clinical staging (Table 1), which supports, but does not prove, the representativeness of this subset. In addition, the exclusion of tumours that were not measurable on CT may have favoured larger and more conspicuous lesions. Third, the cohort comprised only squamous cell carcinomas, with few oral cavity tumours (n = 8) and no other types of oral or head and neck malignancies, and p16 status was not consistently available; the findings may therefore not be generalisable to these tumours.

Fourth, observer agreement could only be partly characterised. CT-GTV was delineated by a single reader (the ENT surgeon, under the supervision of a radiation oncologist), so that the variability of the reference itself was not quantified, although substantial interobserver variability in GTV delineation has been demonstrated in HNC [24]; CT-GTV should therefore be regarded as a clinical reference rather than a gold standard. Moreover, MTV–GTV concordance was assessed on volumes only; spatial overlap metrics (e.g., Dice coefficient or Hausdorff distance) were not computed. Likewise, the two-reader design addresses a specific and clinically relevant question (delegation feasibility) but does not permit formal separation of a generalisable effect of PET segmentation experience from a reader-pair-specific one. Fifth, PET Edge+ contours could be edited with manual grow/shrink tools whereas the threshold methods were not, which introduces an asymmetry between methods. Finally, prognostic outcomes were not addressed in the present work, as they are the subject of the separate report mentioned above.

Future work should therefore include prospective multicentre validation of these findings in harmonised datasets; testing across different PET/CT scanners, reconstruction algorithms and segmentation platforms; multi-reader studies spanning a broader range of PET segmentation experience, with a pre-specified protocol for manual correction; multi-observer delineation of the CT-GTV reference; and evaluation of fully automated deep-learning segmentation. Beyond the separate prognostic analysis currently under review, linking each segmentation method to clinical outcomes in independent cohorts will be necessary to determine which method provides the most prognostically informative MTV.

## 5. Conclusions

In this retrospective single-centre study of 154 patients with primary HNSCC, all four PET segmentation methods available in MIM Maestro Software showed excellent intra- and interobserver volumetric reproducibility. The methods nonetheless differed in two respects. PET Edge+, the only method requiring operator seed placement, was the most precise when used by the reader experienced in PET segmentation; in this reader pair, it was also the only method for which reader experience was associated with a significant method × reader interaction, an effect confined to small-volume tumours (below approximately 15 mL). Agreement with CT-defined GTV was closest for SUV4.0 and weakest for SUV41%. These results do not support a single universal segmentation standard; rather, the segmentation method should be chosen according to its intended use and then applied consistently, and PET segmentation experience should additionally be taken into account when PET Edge+ is used to segment small-volume tumours. Confirmation in prospective multicentre studies across different scanners and software platforms is required before these findings can be generalised.

## Figures and Tables

**Figure 1:**
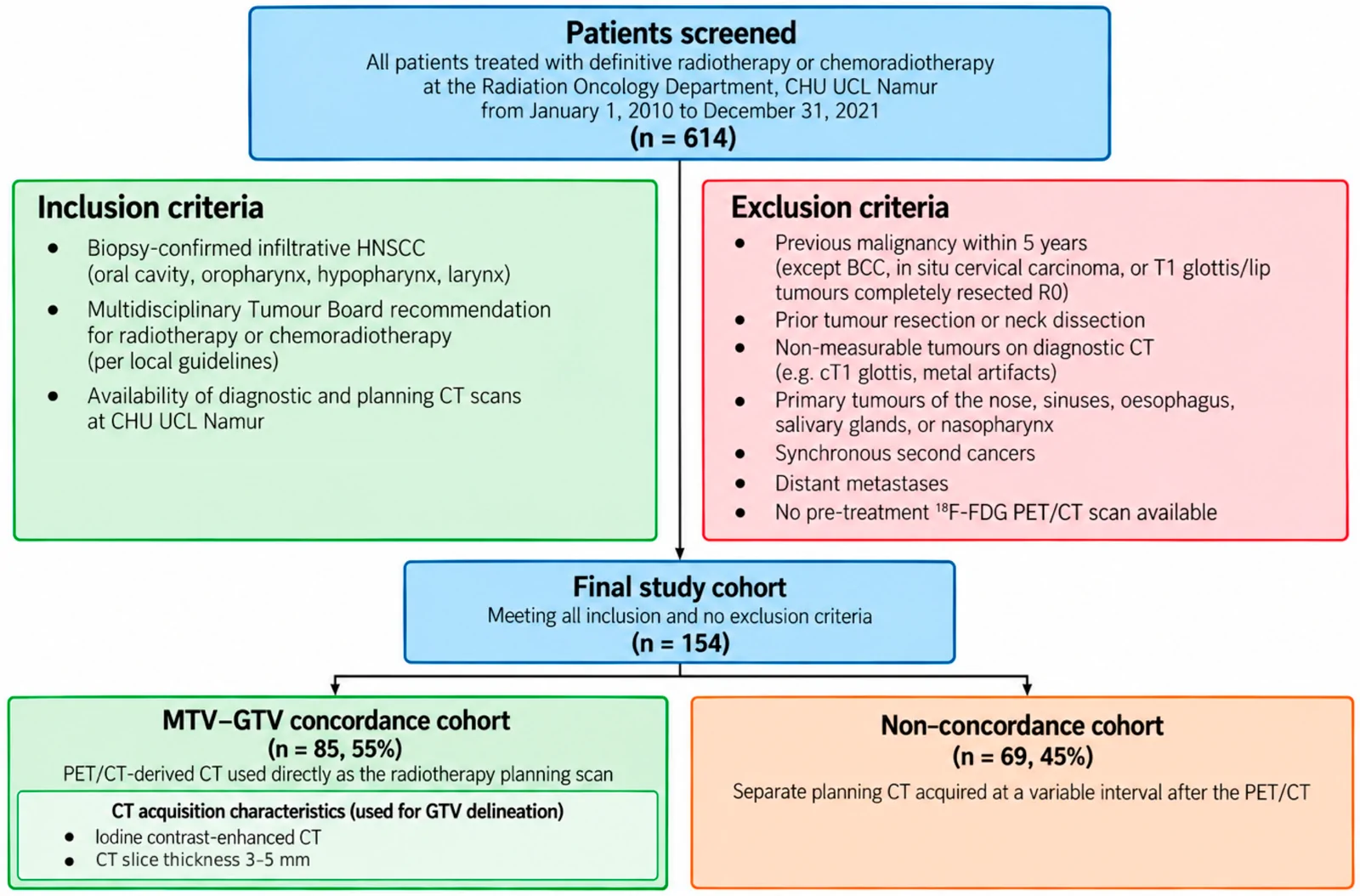
STROBE flow diagram of patient selection. Of the 614 patients screened, 154 met all inclusion criteria and no exclusion criterion; the 85 patients whose PET/CT-derived CT served directly as the radiotherapy planning scan formed the MTV–GTV concordance cohort, and the remaining 69 patients formed the non-concordance cohort.

**Figure 3:**
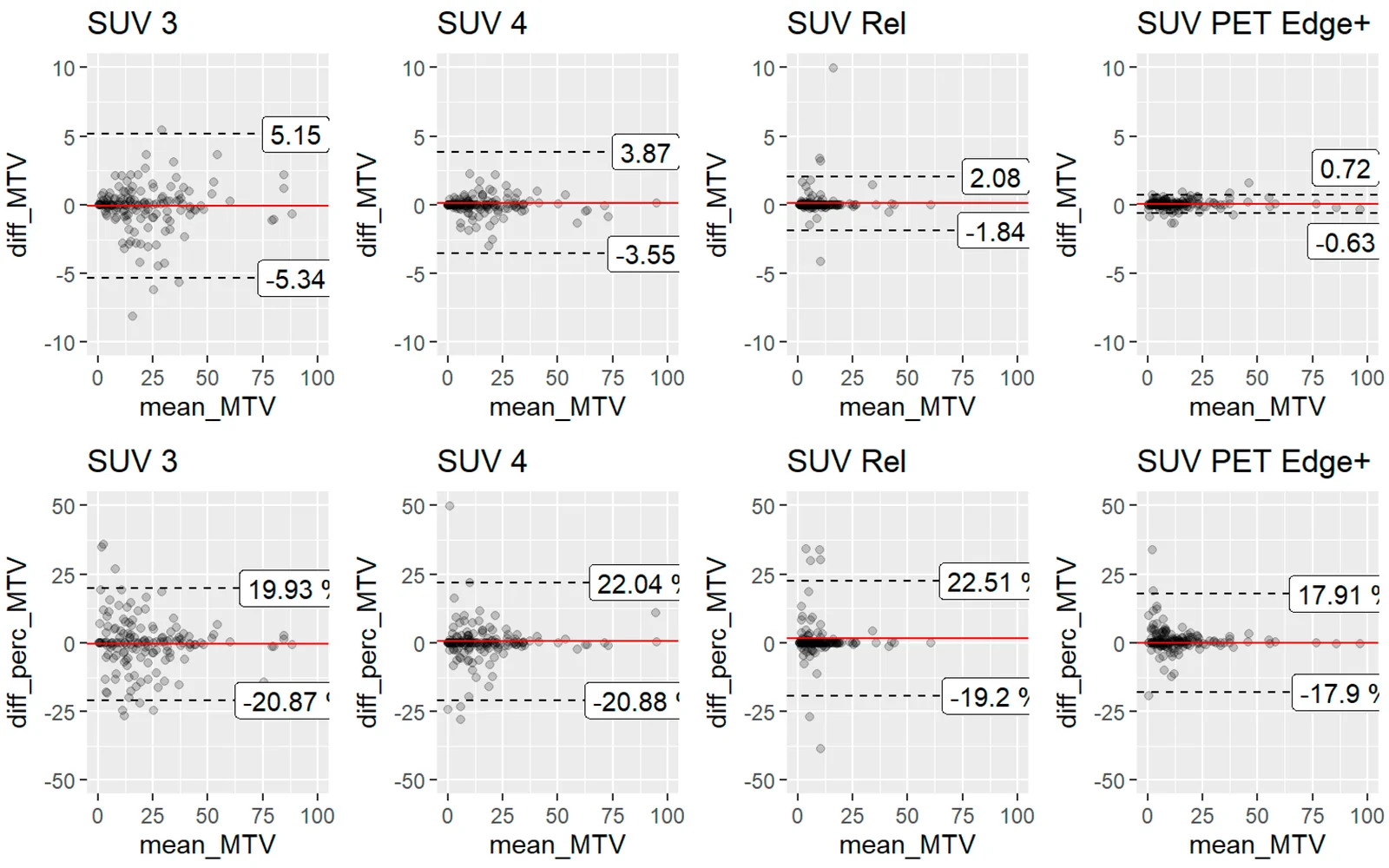
Bland–Altman plots for intraobserver agreement (R1 Session 1 vs. Session 2)—absolute differences (mL) and relative percentage differences. Annotated values are 95% limits of agreement.

**Figure 4:**
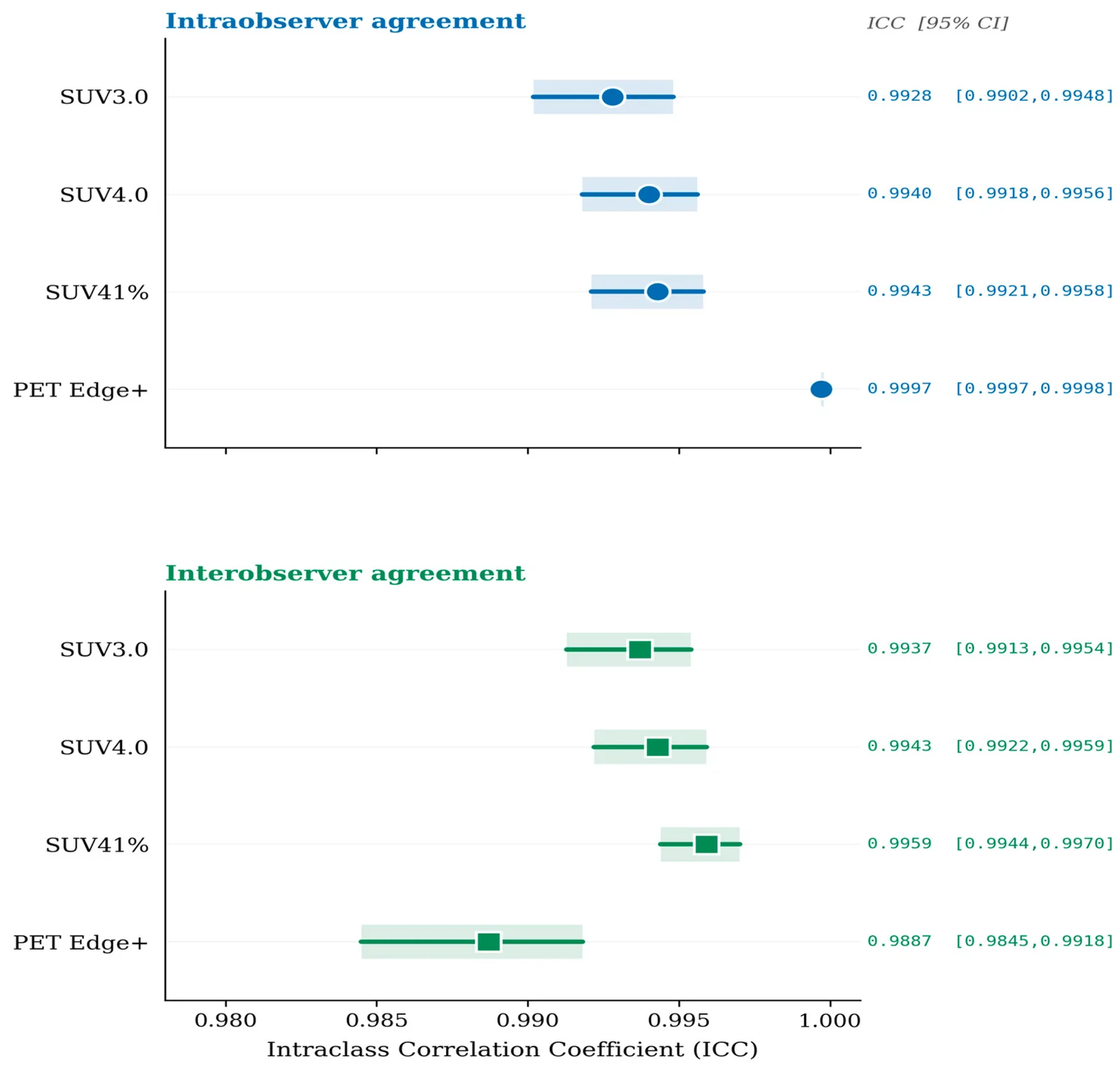
Forest plot of ICC values with 95% confidence intervals, by segmentation method and agreement type. N = 154. All values exceed the threshold for excellent agreement (ICC ≥ 0.90).

**Figure 5:**
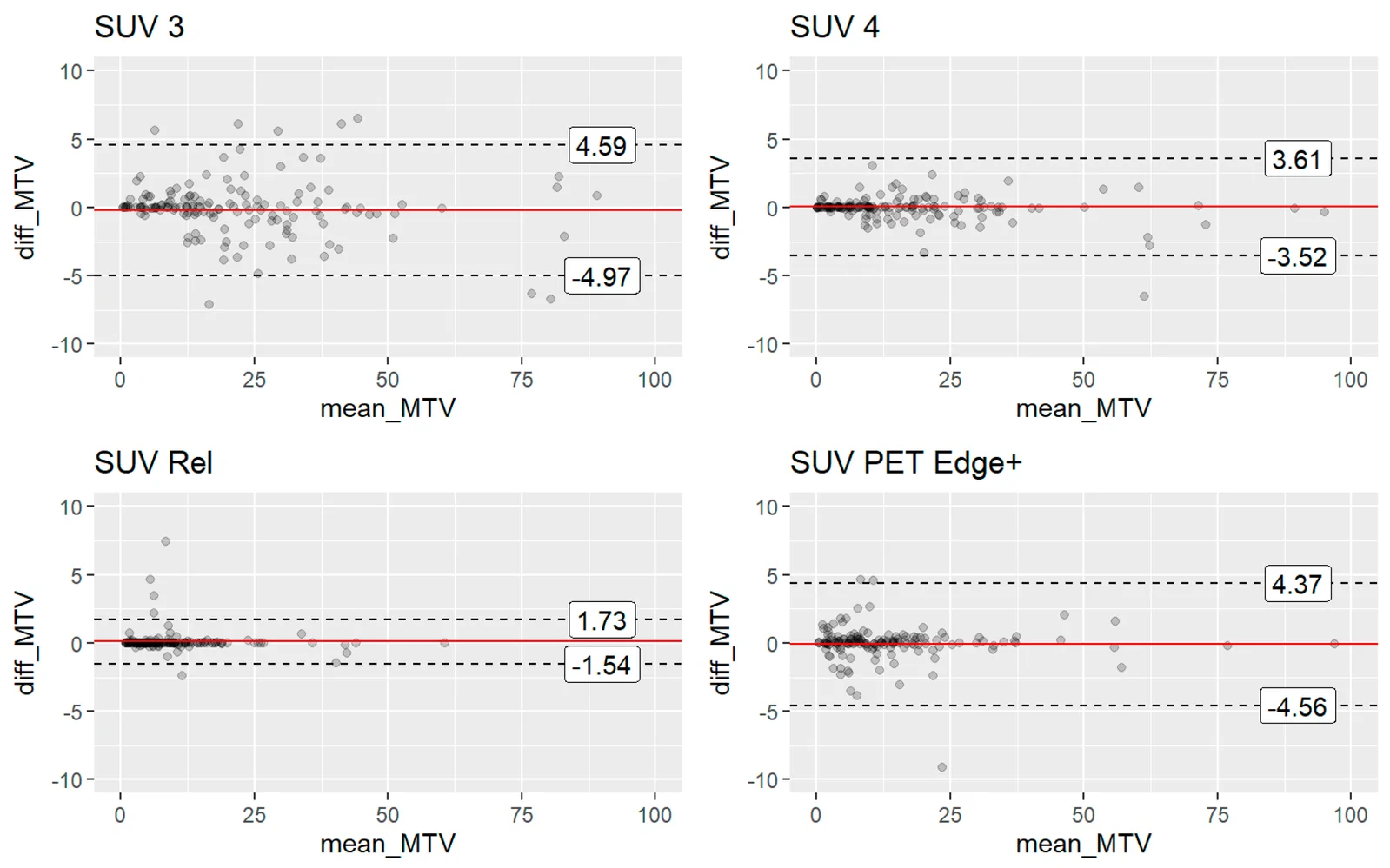
Bland–Altman plots for interobserver agreement (R1 Session 2 vs. R2)—absolute and relative differences.

**Figure 6:**
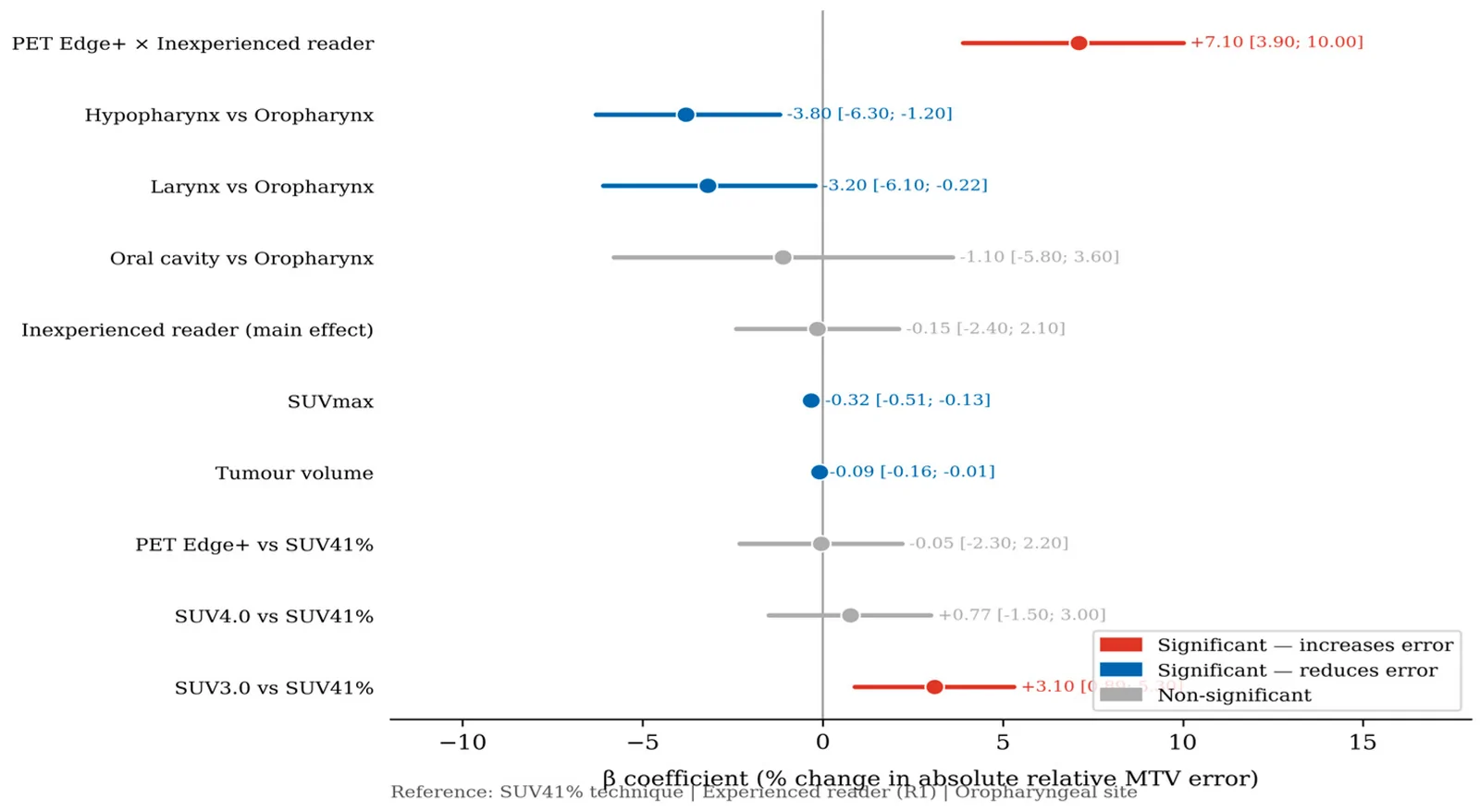
Multivariable regression coefficients (β) for predictors of absolute relative MTV error (%). Reference: SUV41% technique; reader experienced in PET segmentation (R1); oropharyngeal site. Error computed with respect to R1 Session 2. Linear mixed-effects model with a random intercept per patient; N = 1232 observations from 154 patients.

**Figure 7:**
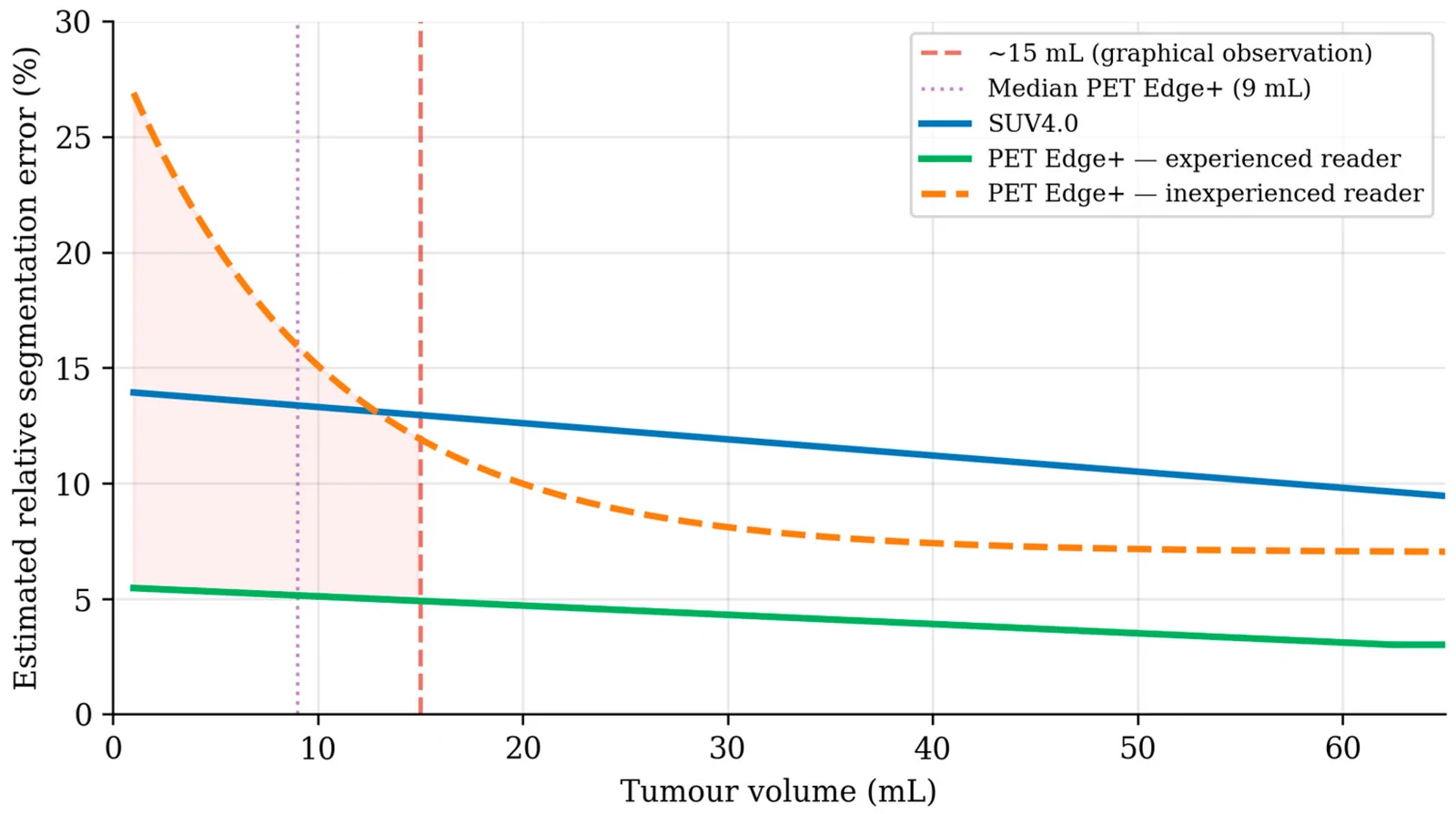
Schematic representation of estimated segmentation error by tumour volume and PET segmentation experience of the reader for PET Edge+ vs. SUV4.0. Visual inspection of the regression-predicted curves suggests a transition around 15 mL, above which performance differences appear negligible regardless of PET segmentation experience. This is a graphical observation, not a formally tested cut-off. Median PET Edge+ volume in the full cohort was 9 mL (purple dotted line).

**Figure 8:**
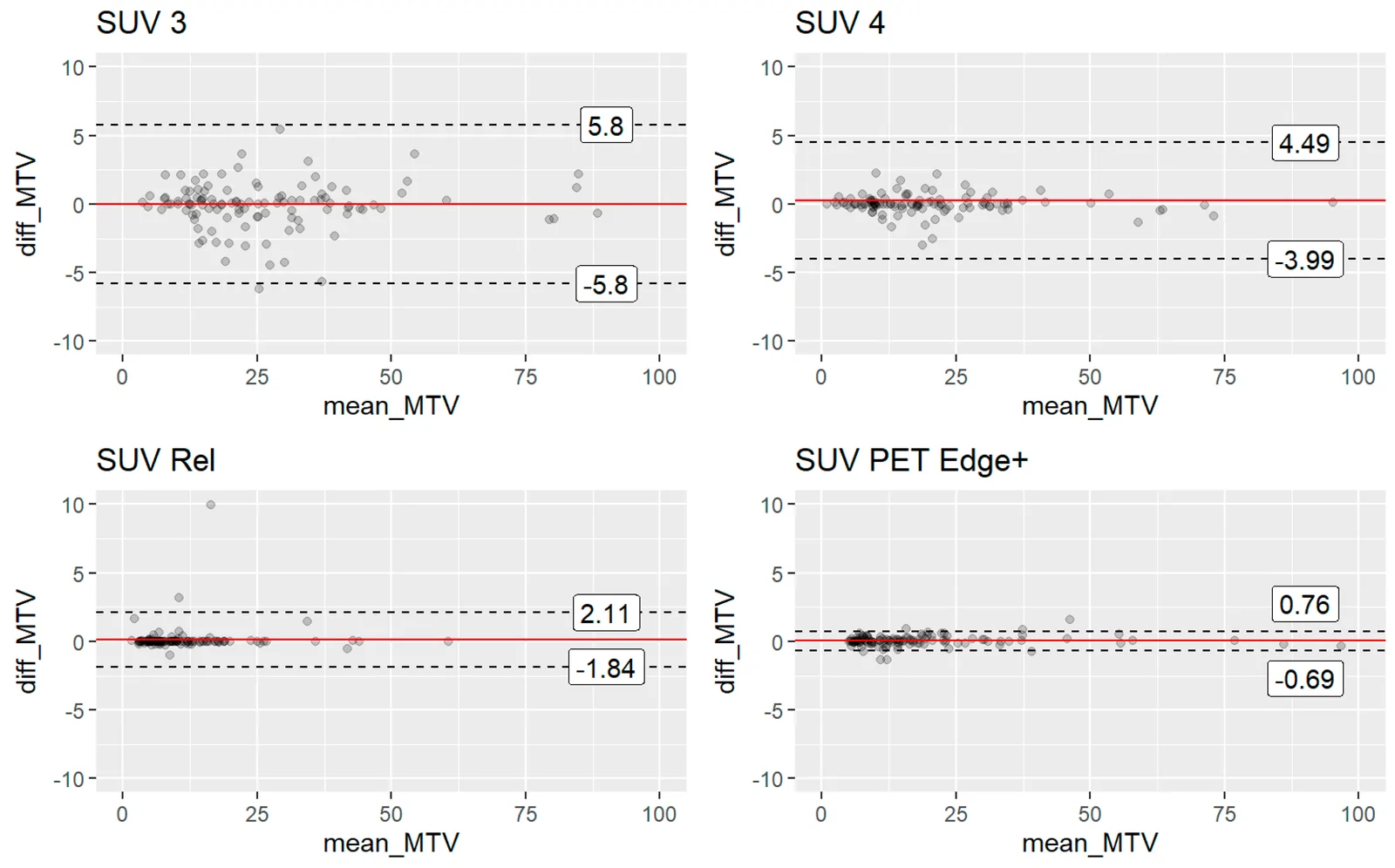
Bland–Altman plots for MTV (R1, Session 2) vs. CT-GTV (R2, n = 85)—absolute differences (mL). Annotated: mean bias and 95% limits of agreement. SUV4.0 shows the closest calibration to GTV (mean bias −1.2 mL).

**Figure 9:**
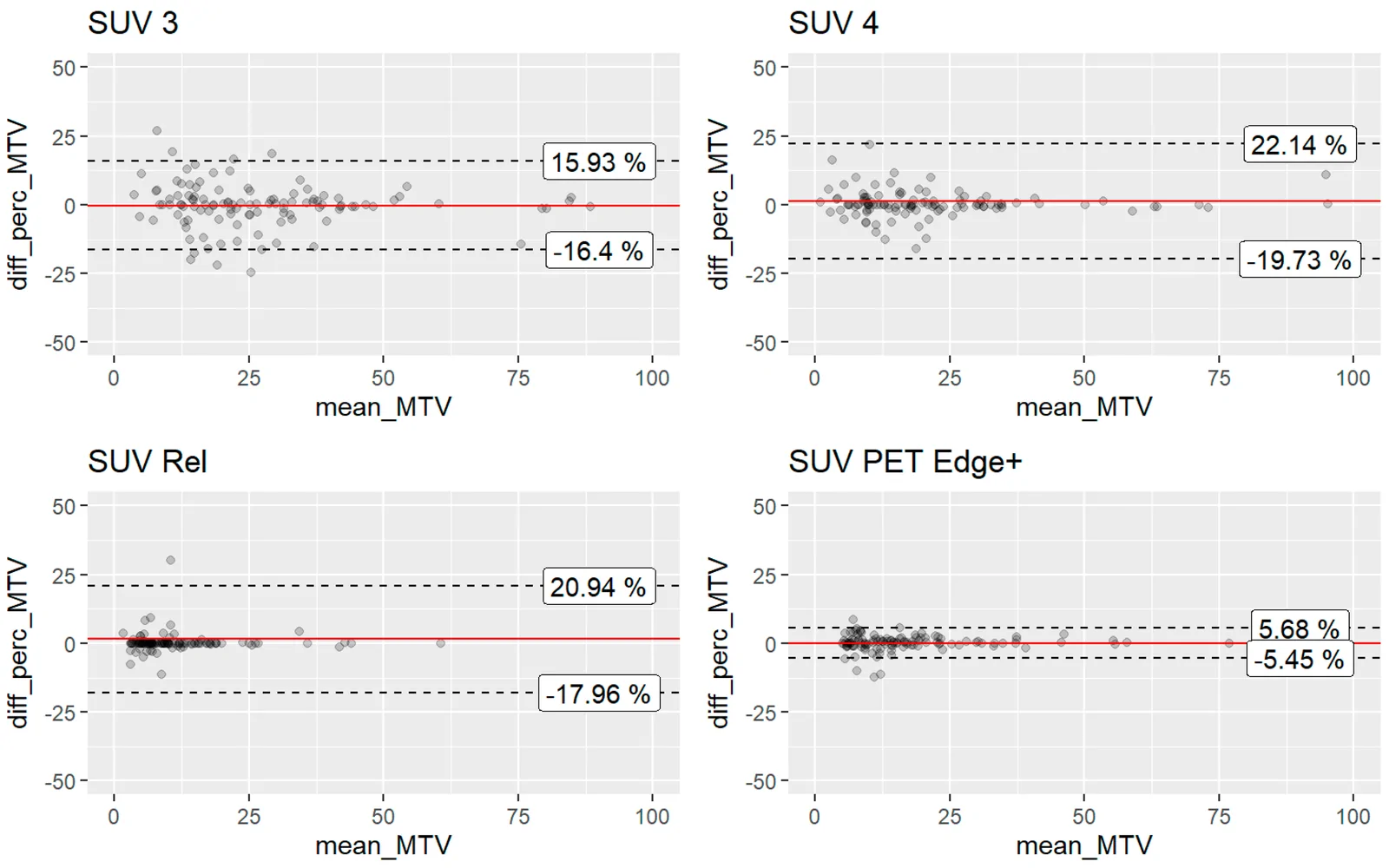
Bland–Altman plots for MTV (R1, Session 2) vs. CT-GTV—relative percentage differences. Mean biases: SUV3.0 +33%; SUV4.0 −6%; SUV41% −48%; PET Edge+ −25%.

**Table 3:** Agreement for MTV by method (N = 154): ICC, within-subject coefficient of variation (CV) and repeatability coefficient (RC). Intraobserver: R1 Session 1 vs. Session 2; interobserver: R1 Session 2 vs. R2. 95% CI from Fisher z-transformation. All ICC values are graded excellent (≥0.90).

Method	Intra ICC [95% CI]	Intra CV (%)	Intra RC (mL)	Inter ICC [95% CI]	Inter CV (%)	Inter RC (mL)
**SUV3.0**	0.993 [0.990, 0.995]	8.1	5.23	0.994 [0.991, 0.995]	7.3	4.78
**SUV4.0**	0.994 [0.992, 0.996]	7.9	3.71	0.994 [0.992, 0.996]	7.5	3.56
**SUV41%**	0.994 [0.992, 0.996]	7.4	1.97	0.996 [0.994, 0.997]	6.2	1.64
**PET Edge+**	>0.999 [>0.999, >0.999]	1.7	0.68	0.989 [0.984, 0.992]	11.3	4.46

**Table 4:** Multivariable linear mixed-effects regression (random intercept per patient) on absolute relative MTV error (%) with respect to R1 Session 2. Reference: SUV41% technique; reader experienced in PET segmentation (R1); “inexperienced reader” = R2, without prior PET segmentation experience; oropharyngeal site. NS = non-significant. ** *p* < 0.01; *** *p* < 0.001. N observations = 1232 (154 patients).

Variable	Beta (%)	95% CI
Technique: SUV41% (reference)	—	—
Technique: SUV4.0 vs. SUV41% (NS)	+0.77	−1.5, 3.0
Technique: SUV3.0 vs. SUV41% **	+3.1	0.89, 5.3
Technique: PET Edge+ vs. SUV41% (NS)	−0.05	−2.3, 2.2
Inexperienced vs. experienced reader—main effect (NS)	−0.15	−2.4, 2.1
Tumour volume—per mL	−0.09	−0.16, −0.01
SUVmax—per unit	−0.32	−0.51, −0.13
Site: hypopharynx vs. oropharynx (reference)	−3.8	−6.3, −1.2
Site: larynx vs. oropharynx	−3.2	−6.1, −0.22
Site: oral cavity vs. oropharynx (NS)	−1.1	−5.8, 3.6
Interaction: PET Edge+ × Inexperienced reader ***	+7.1	3.9, 10
Interaction: SUV4.0 × Inexperienced reader (NS)	+0.70	−2.4, 3.8
Interaction: SUV3.0 × Inexperienced reader (NS)	+0.75	−2.4, 3.9

**Table 5:** Concordance between MTV (R1, Session 2) and CT-GTV (n = 85). GTV discordance: β from regression of |GTV − MTV|/GTV × 100%, reference SUV41%. NS = non-significant; * *p* < 0.05; ** *p* < 0.01.

Comparison vs. CT-GTV	ICC [95% CI]	Grade	Rank	Mean Bias (mL)/GTV Discordance
GTV vs. SUV4.0	0.895 [0.858, 0.922]	Good	1st	−1.2 mL|−16% vs. SUV41% **
GTV vs. PET Edge+	0.846 [0.734, 0.904]	Good	2nd	−5.2 mL|−10% vs. SUV41% *
GTV vs. SUV3.0	0.808 [0.658, 0.883]	Good	3rd	+7.2 mL|+2.2% vs. SUV41% NS
GTV vs. SUV41%	0.572 [0.050, 0.791]	Moderate	4th	−10.2 mL|Reference

## Data Availability

The datasets generated and analysed during the current study are not publicly available due to institutional data governance requirements and patient privacy regulations, but are available from the corresponding author upon reasonable request.
